# Silicon-photonics-enabled chip-based 3D printer

**DOI:** 10.1038/s41377-024-01478-2

**Published:** 2024-06-06

**Authors:** Sabrina Corsetti, Milica Notaros, Tal Sneh, Alex Stafford, Zachariah A. Page, Jelena Notaros

**Affiliations:** 1 Research Laboratory of Electronics, Massachusetts Institute of Technology, Cambridge, MA 02139 USA; 2 Department of Chemistry, The University of Texas at Austin, Austin, TX 78712 USA

**Keywords:** Silicon photonics, Integrated optics

## Abstract

Imagine if it were possible to create 3D objects in the palm of your hand within seconds using only a single photonic chip. Although 3D printing has revolutionized the way we create in nearly every aspect of modern society, current 3D printers rely on large and complex mechanical systems to enable layer-by-layer addition of material. This limits print speed, resolution, portability, form factor, and material complexity. Although there have been recent efforts in developing novel photocuring-based 3D printers that utilize light to transform matter from liquid resins to solid objects using advanced methods, they remain reliant on bulky and complex mechanical systems. To address these limitations, we combine the fields of silicon photonics and photochemistry to propose the first chip-based 3D printer. The proposed system consists of only a single millimeter-scale photonic chip without any moving parts that emits reconfigurable visible-light holograms up into a simple stationary resin well to enable non-mechanical 3D printing. Furthermore, we experimentally demonstrate a stereolithography-inspired proof-of-concept version of the chip-based 3D printer using a visible-light beam-steering integrated optical phased array and visible-light-curable resin, showing 3D printing using a chip-based system for the first time. This work demonstrates the first steps towards a highly-compact, portable, and low-cost solution for the next generation of 3D printers.

## Abstract (web summary)

We propose and demonstrate the first chip-based 3D printer, consisting of a silicon-photonics chip that emits non-mechanically-reconfigurable beams into photocurable resin, enabling future compact, portable, and low-cost next-generation 3D printers.

## Graphical abstract

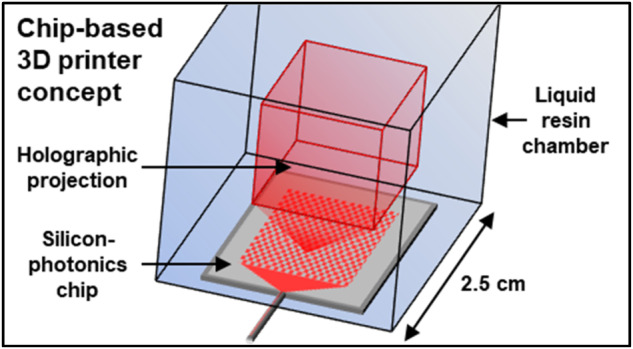

## Introduction

3D printing has transformed modern manufacturing by enabling technologies impacting numerous markets from consumer products to public infrastructure and medicine^1–8^. In recent years, the field has evolved to enable a wide range of print modalities, from high-resolution prints with feature sizes as low as 25 nm^9^ to the fabrication of large-scale components, spanning rocket engines to bridges^8,10^. As the field of 3D printing has grown to encompass an ever-larger application space, researchers have continued to push the boundaries of the field, developing novel methods for printing.

To date, numerous methods have been commercialized for 3D printing using extrusion, powder-bed fusion, jetting, and light-induced polymerization, among other techniques^11^. At the consumer level, fused deposition modeling (FDM), an extrusion-based method, is the most widely used type of 3D printing^12^. In FDM, parts are built layer by layer by heating and extruding thermoplastic filaments^11,13^. While FDM has enabled numerous advances in additive manufacturing, especially in regards to rapid prototyping and bioprinting^14,15^, its filament-based approach tends to result in a lower print resolution compared to other common printing methods^16^.

**Fig. 1:**
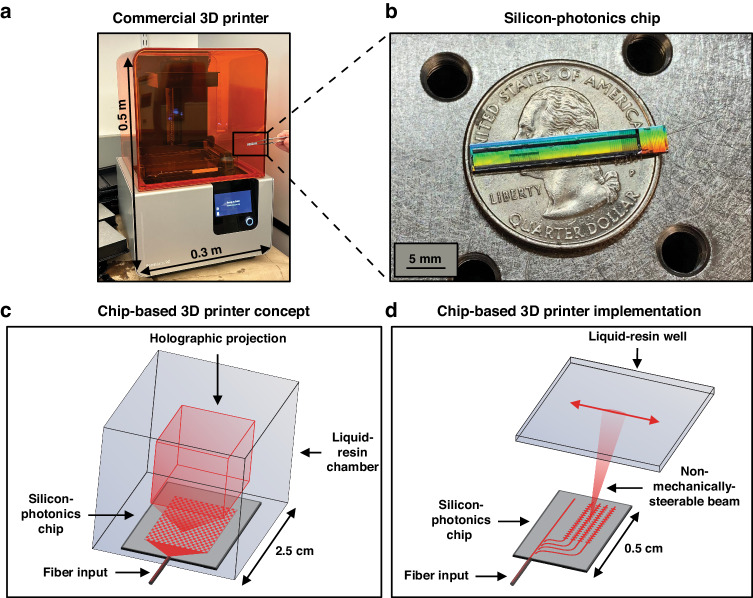
The chip-based 3D printer concept. Photographs showing **a** a typical commercial 3D printer with a photonic chip (outlined in black) for scale and **b** a fabricated and packaged photonic chip. **c** Conceptual diagram of the proposed chip-based 3D printer, showing a hologram formed by a chip within a resin chamber (not to scale). **d** Conceptual diagram of the proof-of-concept stereolithography-inspired chip-based 3D printer demonstrated in this work (not to scale)

In contrast, photocuring-based methods, such as stereolithography (SLA), digital light processing (DLP), and masked stereolithography (MSLA or LCD), offer higher print resolutions, with commercial printers offering feature sizes as low as 10 μm^16^. In SLA, a laser beam is used to solidify a pattern into a thin resin layer on a build platform, after which the build platform lifts and a new layer of liquid resin forms. These cycles of curing and platform movement are repeated layer by layer to form a solid 3D object^11,16,17^. Similarly, DLP printers also create prints through the layer-by-layer curing of resins. However, while SLA creates prints using a single beam, DLP utilizes the projection of entire 2D images onto a resin’s surface, resulting in faster curing^11,16,17^. Although these photocuring-based methods enable increased print resolution, they require bulky and complex mechanical systems (Fig. 1a), including advanced laser routing schemes (for SLA), anti-aliasing measures (for DLP), specialized build platforms, and precise elevator mechanisms^11,16–18^. This requirement for large and complex mechanical systems and reliance on layer-by-layer printing limits portability, form factor, resolution, print speed, and material complexity. To combat these challenges, numerous research efforts have been made to push towards new volumetric modalities for 3D printing that benefit from increased build speeds, a lack of reliance on elevator mechanisms, and decreased anisotropy^19–21^. However, these methods still remain reliant on bulky and intricate mechanical systems, such as rotating stages and multi-angle illumination frameworks. Thus, there remains a growing need for a new compact, portable, and low-cost 3D-printing technology.

The field of silicon photonics has the potential to enable a paradigm-shifting solution to address this need for a next-generation 3D-printing technology. By leveraging scalable CMOS fabrication techniques to enable chip-based optical microsystems with new functionalities, improved system performance, decreased cost, and reduced size, weight, and power, silicon photonics has enabled next-generation optical technologies that have facilitated revolutionary advances for numerous fields spanning science and engineering, including computing, communications, sensing, and quantum engineering^22–29^. An emerging class of integrated photonic systems is integrated optical phased arrays, which consist of an array of on-chip optical antennas fed with controlled phases and amplitudes using an integrated photonic circuit, enabling emission and dynamic control of free-space radiated light in a compact form factor, at low costs, and in a non-mechanical way^30–43^. As such, optical-phased-array-based systems have already emerged as a prominent and promising solution for next-generation LiDAR sensors for autonomous vehicles^29–31^. However, motivated by this initial LiDAR application, integrated-optical-phased-array demonstrations to date have primarily focused on systems that operate at infrared wavelengths, rendering them incompatible with the UV-wavelength-activated photochemistry traditionally used for 3D printing^44^. Integrated optical phased arrays, and the field of silicon photonics in general, have never before been proposed or demonstrated as a solution for 3D printing.

In this paper, to address this need for an advanced 3D-printing technology, we combine the fields of silicon photonics and photochemistry to propose the first chip-based 3D printer. The proposed system consists of only a single millimeter-scale photonic chip without any moving parts that emits reconfigurable visible-light holograms up into a simple stationary resin well to enable non-mechanical 3D printing (Fig. 1c). It presents a highly-compact, portable, and low-cost solution for the next generation of 3D printers.

First, we propose this general chip-based 3D printer concept and outline the key requirements for a complete implementation. Second, as a proof-of-concept demonstration, we experimentally demonstrate a stereolithography-inspired version of this chip-based 3D printer concept by combining the emerging technologies of visible-light integrated optical phased arrays and visible-light-activated photochemistry; the system consists of a visible-light integrated optical phased array that emits and non-mechanically steers a beam up into a well of visible-light-curable resin. Third, we utilize this system to photocure a voxel, thus demonstrating 3D printing using a chip-based system for the first time. Fourth, we characterize the curing rate of this system by measuring the size of individual 3D-printed voxels as a function of curing time, observing printing of sub-millimeter-scale voxels within seconds. Fifth, we utilize the non-mechanical beam-steering capabilities of the system to implement 3D printing of lines in one dimension without any moving parts. Finally, we extend this capability to demonstrate 3D printing of arbitrary patterns in two dimensions using the system.

## Results

### The silicon-photonics-enabled chip-based 3D printer concept

The proposed chip-based 3D-printing system consists of a single flat-form-factor millimeter-scale silicon-photonics chip sitting at the bottom of a simple stationary well of visible-light-curable liquid resin, as shown in Fig. 1c. The proposed chip projects visible-light 3D holograms in the shape of a desired object upwards into the resin well to induce selective solidification of the resin, resulting in a volumetric 3D print. By eliminating the need for elaborate mechanical build platforms and laser-routing systems, our proposed system will enable a shift in standard operating conditions for 3D printers, transitioning from benchtop setups with bulky form factors to a portable chip-based system with a hand-held form factor. To implement such a system, several key components are required.

The system involves a chamber of liquid resin designed to cure selectively and rapidly when exposed to the wavelength of light emitted from the silicon-photonics chip^45^. Since silicon-photonics systems generally operate at infrared or visible wavelengths to ensure reasonable waveguiding material losses and device feature sizes compatible with chip fabrication limitations, the resin is designed to cure at visible wavelengths, as opposed to the shorter UV wavelengths typically used to activate commercial resins. The resin is modular, with the ability to replace the components of the photosystem as required for different wavelengths depending on the desired application^45,46^. In the proposed system, a silicon-photonics chip sits at the bottom of this chamber and projects a reconfigurable programmable visible-light hologram upwards into the resin, causing the resin to selectively polymerize into a solid print in the shape of the desired object.

The creation of holographic images requires the ability to precisely tune the local phase and amplitude of the light emitted from an aperture^47,48^. As such, the proposed system incorporates a 2D grid of on-chip visible-light integrated optical phased arrays that act as the pixels of the aperture, akin to the system demonstrated in^48^, previously developed for applications in visible-light holographic displays for augmented reality. These optical-phased-array-based pixels are encoded to emit light with the appropriate amplitudes and phases such that a holographic image is formed. Specifically, the phase and amplitude distributions necessary for generating a desired holographic image are closely approximated by discretizing these ideal continuous distributions into local one-dimensional phase gradients with arbitrary amplitudes and absolute phases corresponding to the size of the pixels in the aperture. These discretized distributions are then iteratively optimized using the Gerchberg-Saxton algorithm^49,50^ to accurately generate the desired holographic image. The resulting amplitude, absolute-phase, and phase-gradient encodings for each optical-phased-array-based pixel are then applied using a set of integrated modulators.

To enable routing and emission of light at visible wavelengths, critical for ensuring compatibility with the visible-light-curable resin, the system is based on silicon-nitride waveguides, since silicon nitride has a low absorption coefficient within the visible spectrum and is CMOS compatible. However, silicon nitride has a low thermo-optic coefficient and does not exhibit significant electro-optic properties, which has made integrated modulation at visible wavelengths challenging^51,52^. As such, liquid-crystal material, with a strong birefringence in the visible spectrum, is integrated into the silicon-photonics platform and used to enable dynamic modulation and encoding of the optical-phased-array-based pixels in the system, as demonstrated in^53–55^.

The proposed system is designed to be heterogeneously integrated with a co-designed CMOS electronics chip^30,33,34^ to enable the electronics control necessary for driving the modulators while maintaining the compact form factor of the chip-based 3D printer.

In the following sections, we develop a proof-of-concept system that serves as a fundamental stepping stone on the path towards this volumetric chip-based 3D-printer vision. Specifically, we experimentally demonstrate a stereolithography-inspired version of the chip-based 3D printer (Fig. 1b, d) capable of 3D printing arbitrary patterns in two dimensions; the system consists of a visible-light integrated optical phased array that emits and non-mechanically steers a beam up into a well of visible-light-curable resin.

### Visible-light integrated optical phased array system

**Fig. 2:**
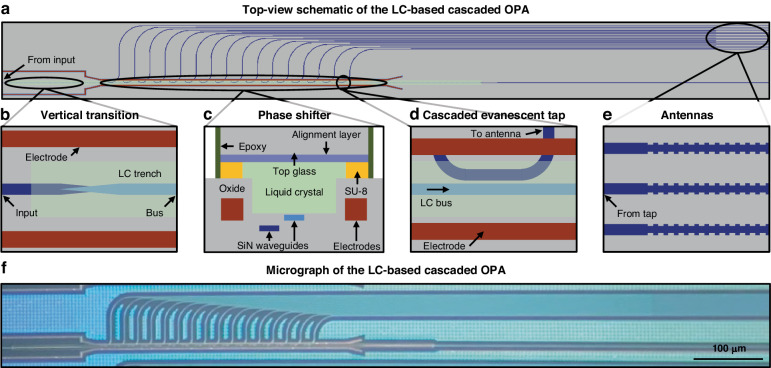
The 3D-printer integrated optical phased array architecture^53,54^. **a** Top-view simplified schematic of the visible-light liquid-crystal-based cascaded integrated optical phased array that enables the stereolithography-inspired chip-based 3D-printer system. **b** Top-view simplified schematic of the vertical-transition escalator from the bottom waveguide to the top waveguide directly underneath the liquid-crystal region (not to scale). **c** Cross-sectional simplified diagram of the phase shifter after the in-house post-processing packaging steps (not to scale). **d** Top-view simplified schematic of a cascaded evanescent tap that couples light from the upper bus waveguide to the bottom tap waveguide (not to scale). **e** Top-view schematic of the grating-based antennas (not to scale). **f** Micrograph of the fabricated and packaged visible-light liquid-crystal-based cascaded integrated optical phased array

As a proof-of-concept demonstration, we develop and experimentally demonstrate a stereolithography-inspired version of the chip-based 3D printer. The silicon-photonics chip is based on a visible-light integrated optical phased array consisting of a liquid-crystal-based cascaded-phase-shifter architecture that linearly controls the relative phase applied to an array of antennas, as shown in Fig. 2a and as previously developed in^53,54^. In this paper, we provide key details regarding the visible-light integrated optical phased array system in the context of the stereolithography-inspired chip-based 3D printer. Additional details regarding the visible-light integrated optical phased array are provided in^53,54^.

At the input, an on-chip inverse-taper edge coupler couples 637-nm-wavelength light from an off-chip laser into an on-chip single-mode 160-nm-thick silicon-nitride waveguide. A 100-µm-long escalator device (an adiabatic layer-transition structure) then couples the input light from the single-mode waveguide into a second 160-nm-thick silicon-nitride bus waveguide that is 10 nm below a liquid-crystal-filled trench (Fig. 2b). Next, evanescent tap couplers, placed with a pitch of 20 µm and with increasing coupling lengths, uniformly distribute the light from the bus waveguide to 16 vertically stacked and horizontally offset tap waveguides (Fig. 2d), as detailed in^54^. These tap waveguides then route to 16 grating-based uniform-perturbation 400-µm-long antennas with a 2-µm pitch to emit the light out of the surface of the chip with an exponential emission profile (Fig. 2e), as simulated in^54^.

To enable non-mechanical beam steering, phase modulation of the light emitted out of the array of antennas is required. However, modulation at the visible wavelengths required for this system is difficult because silicon nitride has a low thermo-optic coefficient and does not exhibit significant electro-optic properties^51,52^. Thus, to enable one-dimensional far-field beam steering, the system leverages the birefringence of liquid-crystal medium to enable cascaded phase control to the array of antennas. In a nematic liquid-crystal medium, the refractive index varies based on the orientation of the liquid-crystal molecules with respect to the propagation direction of the light. Thus, by applying an electric field across the liquid-crystal region to orient the molecules in the direction of the applied field, the index of the liquid-crystal media can be actively tuned, resulting in a change in the effective refractive index of the optical mode in the waveguide and a linear phase shift to the antennas, as simulated in^54^. To enable this functionality, the liquid-crystal-based phase-shifting region consists of a silicon-nitride bus waveguide to weakly confine and guide the light, liquid-crystal medium deposited into a silicon-dioxide trench to enable strong interaction between the optical mode and the liquid-crystal medium, metal electrodes on each side of the liquid-crystal-filled trench for applying an electric field across the liquid-crystal region, and a top glass chip with a mechanical alignment layer on the underside to anchor the liquid-crystal molecules. A cross-sectional diagram of the phase-shifting region is shown in Fig. 2c. Additional details regarding the integrated optical phased array are provided in^53,54^.

This liquid-crystal-based cascaded integrated optical phased array was fabricated in a CMOS-compatible 300-mm wafer-scale silicon-photonics process at the State University of New York Polytechnic Institute’s (SUNY Poly) Albany NanoTech Complex. We then diced the fabricated photonics wafer and performed a chip-scale liquid-crystal-packaging process at MIT. A micrograph of the fabricated and packaged integrated optical phased array is shown in Fig. 2f. Further details on the wafer fabrication and liquid-crystal packaging are provided in the Materials and Methods section.

### Visible-light-curable liquid resin

While typical commercial resins cure when exposed to UV wavelengths, these typical resins are incompatible with curing using silicon-photonics chips, since silicon-photonics systems generally operate at infrared or visible wavelengths to ensure reasonable waveguiding material losses and device feature sizes compatible with chip fabrication limitations. Therefore, moving to longer visible or infrared curing wavelengths is necessary for compatibility with silicon photonics. However, developing resins that cure at longer wavelengths is challenging, hence the UV-activation standard for commercial resins. Therefore, the resin used in this stereolithography-inspired proof-of-concept demonstration of the chip-based 3D printer is a custom three-component-photosystem-based resin designed for efficient photocuring at visible wavelengths, as previously developed in^45,46^.

Typical resin photosystems rely on either a single photoinitiator or a combination of a photocatalyst and either a hydrogen donor or an electron donor/acceptor. In the latter case, photocuring occurs by electron transfer from a photoredox catalyst to a coinitiator, followed by bond scission to generate radicals or ions that initiate polymerization^56–60^. While the use of a photoredox compound enables photocuring via excitation of a π → π* transition with high attenuation at visible wavelengths (>500 nm), photosystems of this kind typically exhibit long curing times (>60 s) due to their multistep reaction mechanisms. To combat this, we use a three-component photosystem in this work^45^, consisting of a photoredox compound and a pair of coinitiators, rather than a single coinitiator. The presence of the two coinitiators enables curing that promotes both photoredox compound regeneration and doubles the concentration of radicals produced per photon absorbed, as detailed in^45^.

To create the resin used in this work, as discussed in detail in the Materials and Methods section, we first synthesize the photoredox compound (aza-Br). During the synthesis process, we halogenate the compound to increase its intersystem crossing rate to long-lived triplet excited states, further improving the reaction efficiency of the photosystem by increasing the number of collisions between initiators and excited photoredox compounds per photon absorbed^61^. The resulting aza-Br compound exhibits peak extinction around 660 nm, enabling compatibility with the chip-based 3D printer’s operating wavelength of 637 nm^46^. To complete the photosystem, we combine the aza-Br with a coinitiator pair of Borate V and H-Nu 254. Finally, we fully dissolve the photosystem in a mixture of tetraethylene glycol diacrylate and trimethylolpropane triacrylate to create the complete resin. Dissolving the photosystem is a straightforward process involving the mixture of the photosystem components into the acrylate. This simple procedure can be performed outside of a chemistry laboratory environment, indicating the practicality of using similar visible-light-activated resins for future iterations of the chip-based 3D printer.

### Beam-forming and 3D-printed voxel results

Using the visible-light integrated optical phased array and the visible-light-curable resin described above, we demonstrate 3D printing using a chip-based system for the first time.

**Fig. 3:**
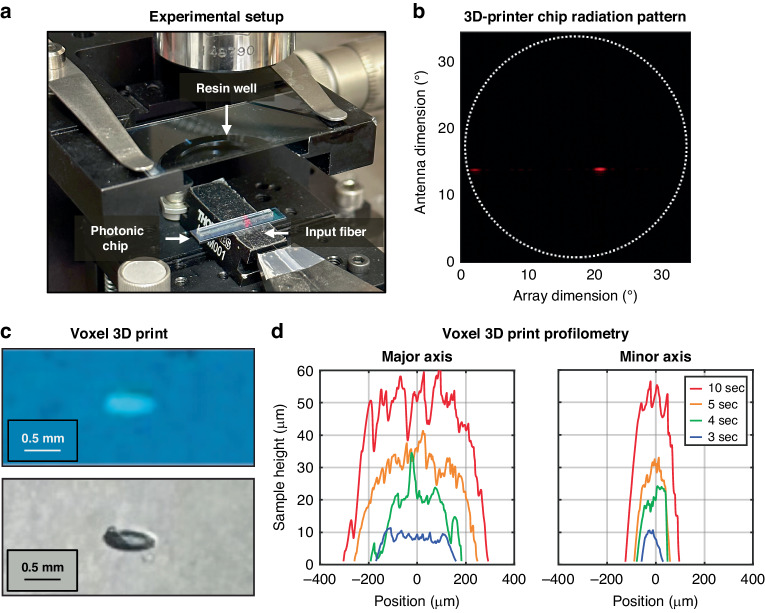
Chip-based 3D printer setup, radiation pattern, and voxel characterization. **a** Photograph of the setup used for the proof-of-concept 3D-printer demonstration, depicting the input fiber, photonic chip, and resin well. **b** Measured far-field radiation pattern emitted by the visible-light integrated optical phased array used for the stereolithography-inspired chip-based 3D printer, showing the main lobe and a grating lobe^53,54^. **c** Photographs of a 3D-printed voxel, created using the chip-based printer, within a well of remaining liquid resin (top) and the same solid 3D-printed voxel after separation from the remaining liquid resin (bottom). **d** Measured dimensions for four separate voxels 3D printed with varying printing times of 3, 4, 5, and 10 s, demonstrating the formation of voxels as a function of time

The setup used for this printing demonstration is shown in Fig. 3a. The fabricated and packaged 3D-printer photonic chip is mounted on a chuck, with a sample stage that supports liquid resin wells mounted on a positioning system above the chip. Light is routed from an off-chip diode laser centered at 637 nm via a P1-630Y cleaved fiber and is edge coupled to the on-chip system. The setup is visualized using a 10x Mitutoyo objective, and split into both near- and far-field imaging paths using a visible beam splitter and focusing lens. Using this setup, we image the beam emitted by the integrated optical phased array in the far field, resulting in the pattern depicted in Fig. 3b. The beam has a power full width at half maximum of 0.4° × 1.6°, closely matching the 0.1° × 1.0° beam size expected for a uniformly emitting 400 µm × 32 µm aperture, with the difference in the antenna dimension attributed to the non-uniform exponential emission pattern of the grating-based antennas. The beam is elliptical due to the difference in aperture size between the antenna and array dimensions; this symmetry could be improved in the future by introducing a cylindrical collimating lens above the chip^62^ or by utilizing an integrated optical phased array with the same aperture size in both dimensions. The overall insertion loss from the on-chip input waveguide to the main lobe emitted by the integrated optical phased array is approximately 25 dB, which could be improved by utilizing strong evanescent tap couplers instead of the weak taps used in this initial demonstration and by reducing the antenna pitch to decrease the power loss to the higher-order grating lobes^54^. Further details regarding integrated-optical-phased-array beam-forming characterization are provided in^53,54^.

To create a well for the liquid resin, two coverslips are separated by one layer of double-sided tape on the left-hand side of the well and one layer of single-sided tape on the right-hand side of the well to ensure that the coverslips are securely attached, while also creating an easy-access hinge for removing cured prints from the well. This creates a chamber with a thickness of approximately 60 µm into which 55 µL of liquid resin is pipetted. The well containing the resin is then clamped into the sample stage above the chip and centered on the main lobe emitted by the integrated optical phased array. Resin preparation and 3D printing are performed in a dark environment to prevent curing from visible-light sources other than the integrated optical phased array.

After centering the resin sample on the main lobe of the integrated optical phased array, we use the visible-light microscope path to monitor curing, which is observable via diffraction of the integrated optical phased array’s radiation pattern around a solidified voxel. As depicted in Fig. 3c, a 3D-printed voxel presents as a bleached region (white/transparent) within remaining leftover resin (blue) due to photobleaching of the blue aza-Br photoredox compound during the photocuring reaction. Using a Kim wipe and isopropyl alcohol, we separate the cured print from the remaining uncured liquid resin. The result is a free-standing 3D-printed voxel in the shape of the integrated optical phased array’s main lobe (Fig. 3b). The single voxel depicted in Fig. 3c measures approximately 0.5 mm × 0.125 mm in the plane of the photograph with a height of approximately 60 μm. The voxel height is determined by the spacing of the resin well’s coverslips (60 μm for this test). The planar dimensions of the voxel are determined by the distance between the integrated optical phased array and the resin well, since the integrated optical phased array used for this proof-of-concept 3D printer emits a diffracting beam (0.4° × 1.6°). In this demonstration, we placed the resin well 2.5 cm over the surface of the 3D-printer chip, resulting in sub-millimeter-scale voxels (Fig. 3c, d). To decrease the divergence of our beam and therefore enable printing of smaller voxels at the same distance from the 3D-printer chip, we could design future iterations of the 3D printer with a larger emitting aperture. Furthermore, the use of focusing integrated optical phased arrays developed by our group^35^, which enable the 3D focusing of emitted light to a tightly confined spot, in future iterations of the chip-based 3D printer would enable printing of even smaller micron-scale voxels, offering a significantly higher print resolution even when compared to the current commercial standard of tens to hundreds of microns^63^.

To characterize the curing rate and print formation process of these voxel prints, we performed curing and profilometry for 3D prints with varying curing time intervals (Fig. 3d). Specifically, we 3D printed four single voxels using four separate resin wells with varying printing times of 3, 4, 5, and 10 s. After separating the resulting voxels from the remaining liquid resin, we used a Veeco Dektak 150 Surface Profilometer to measure the height of the prints along their major and minor (longer and shorter) axes. As depicted in Fig. 3d, we find that 3D-printed voxels grow as a function of time, eventually reaching a plateau upon growing to the top of the resin well, at a height of approximately 60 μm. To demonstrate the rapid curing capability of the system for this test, we set the power of the off-chip diode laser such that approximately 6.7 μW of optical power was supplied to the main lobe of the integrated optical phased array. At this optical power, we observe voxel print formation and adherence to a glass slide within seconds (Fig. 3d). Even at significantly lower optical powers on the order of 100 pW, we can still observe voxel formation within 10 min, with a nonlinear relationship between optical power and printing time^45,46^.

By adjusting either the resin or the silicon-photonics system utilized in the chip-based 3D printer, we could even further improve the print speed. First, we could increase the optical power supplied to the chip by switching to a higher-power laser source, with the expectation that the print speed will increase proportional to the square root of the incident optical intensity^64^; since silicon-nitride waveguides exhibit high power-handling capabilities of up to hundreds of milliwatts and acrylic-based resins frequently operate at intensities larger than 1 mW/cm^2^ in commercial SLA printers, we expect that we could significantly increase the power supplied to the chip without damaging the resins or the chip. Furthermore, we could design future iterations of the chip-based 3D printer for a different operating wavelength. Specifically, we could adjust the wavelength to more closely match the absorption peak of the red-light-activated resin. Alternatively, we could design future iterations for a blue operating wavelength, allowing for compatibility with even more rapidly curable blue-light-activated resins capable of printing at speeds up to 45 mm/h when tested in a commercial DLP printer^45^, competitive with standard commercial print speeds on the order of 100 mm/h. Finally, we could adjust the visible-light-activated photosystem to be compatible with oxygen scavengers or pass argon over samples during printing to minimize oxygen inhibition^65^.

### Non-mechanical beam-steering and 3D-printed pattern results

Utilizing the non-mechanical beam-steering capabilities of the visible-light integrated optical phased array, we now move beyond the printing of single voxels and demonstrate 3D printing of one- and two-dimensional patterns.

**Fig. 4:**
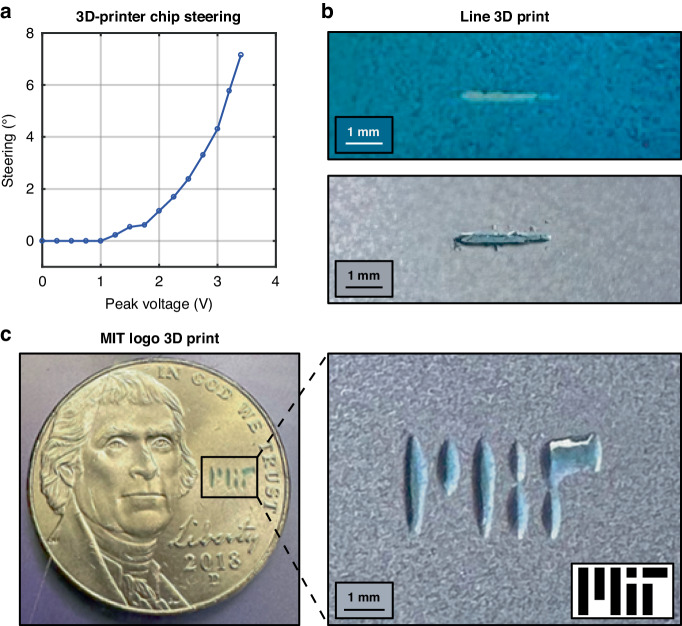
Chip-based 3D printer non-mechanical beam steering, line print, and arbitrary 2D pattern print. **a** Electrically controlled beam steering of the main lobe of the 3D-printer chip’s visible-light integrated optical phased array when a square wave with a varying peak voltage is applied across the liquid-crystal-based phase shifter^53,54^. **b** Photographs of a 3D-printed line, created using the chip-based printer, within a well of remaining liquid resin (top) and the same solid 3D-printed line after separation from the remaining liquid resin (bottom). **c** Photographs of a 3D-printed MIT logo created using the chip-based printer, with a U.S. nickel for scale (left) and zoomed in (right)

To enable non-mechanical beam steering in the array dimension of the integrated optical phased array, we use electronic probes to contact the photonic chip’s integrated electrodes and apply a 10-kHz square wave across the electrodes of the liquid-crystal-based phase shifter. We vary the peak voltage of this applied square wave to tune the phase gradient applied across the antennas and, hence, steer the formed beam in the array dimension by up to 7.2° within ±3.4 V, as shown in Fig. 4a. The beam-steering range is determined by the position of the higher-order grating lobes resulting from the >λ/2 antenna pitch and the maximum attainable liquid-crystal refractive index before the waveguide mode becomes poorly confined. Further details regarding integrated-optical-phased-array beam-steering characterization are provided in^53,54^.

Using this non-mechanical beam-steering capability, we demonstrate 3D printing of a line into the resin, as shown in Fig. 4b. We print this line by sweeping the voltage applied to the liquid-crystal-based phase shifter from 0 V_p_ to 3 V_p_. As the voltage applied to the phase shifter is increased, the refractive index of the liquid crystal relative to the mode in the phase shifter increases, leading to lower confinement of the mode in the bus waveguide. As a result, the power delivered to the integrated optical phased array antennas decreases as the radiation pattern steers. To compensate for this, we print for longer times at higher voltages than for lower voltages, to ensure uniform curing along the line. As depicted in Fig. 4b, the 3D-printed line is distinguishable from the remaining blue liquid resin, as in the case of the single-voxel print in Fig. 3c. We again separate the print from the remaining liquid using a Kim wipe and isopropyl alcohol, and we demonstrate that the result is once again a free-standing 3D-printed solid. For the current proof-of-concept 3D printer, the maximum length of a printed line is determined by the integrated optical phased array’s beam-steering range. For future iterations of the chip-based 3D printer, we could implement integrated optical phased arrays with a decreased antenna pitch; this will increase the distance between the main and higher-order grating lobes emitted by the integrated optical phased array and increase the beam-steering range, thus enabling the printing of significantly larger objects.

Using single-line prints as a building block, we now demonstrate the system’s ability to 3D print arbitrary patterns in two dimensions, thus realizing our proof-of-concept stereolithography-inspired chip-based 3D printer system. To transition from single-line prints to arbitrary two-dimensional patterns, we use the resin well’s mechanical positioning stage. Specifically, after printing each one-dimensional line in a desired pattern, we use the mechanical stage to move the well with micron-scale precision in the second dimension. As a specific demonstration, we perform a print of the Massachusetts Institute of Technology (MIT) logo, creating each line in the print from a subset of the single-line voltage range (0 to 3 V_p_). To promote rapid yet controlled printing for this demonstration, we set the power of the off-chip diode laser such that approximately 1.9 μW of optical power was supplied to the main lobe of the integrated optical phased array. Between each line of the print, we decrease the integrated optical phased array’s output power to prevent curing between components. As oxygen in the sample is quenched during the print, the curing rate becomes faster^45^. We thus adapt the amount of time spent on each component of the logo to ensure uniform curing (e.g., printing the vertical component of the “T” in the logo faster than the bottom component of the “I”). The approximate times to print the three lines of the “M”, the dot and the body of the “I”, and the vertical and horizontal components of the “T” were 1.8, 0.2, 1.4, 0.2, 1.0, 0.7, and 0.5 min, respectively, with a few seconds of switching time allotted between each segment of the print. The final print, performed in under 6 min total, is depicted in Fig. 4d, once again separated from any remaining liquid resin using a Kim wipe and isopropyl alcohol. To further improve our two-dimensional pattern print speed, we could calibrate and automate the voltage sweep applied to the phase shifter, based on the anticipated curing rate at each portion of the print. In addition, we could increase the power of the off-chip diode laser such that we supply >1.9 μW of optical power to the main lobe of the integrated optical phased array; to enable pattern printing at higher optical powers, such as the 6.7-μW main-lobe power utilized for the rapid single-voxel printing demonstration above, we could also automate the on/off switching for the diode laser between segments of the print to prevent undesired curing between separate pattern segments.

## Discussion

This work combines the fields of silicon photonics and photochemistry to propose the first chip-based 3D-printing technology. The proposed system consists of only a single millimeter-scale photonic chip without any moving parts that emits reconfigurable visible-light holograms up into a simple stationary resin well to enable non-mechanical 3D printing.

First, we proposed this general chip-based 3D printer concept and outlined the key requirements for a complete implementation. Second, as a proof-of-concept demonstration, we experimentally demonstrated a stereolithography-inspired version of this chip-based 3D printer concept by combining the emerging technologies of visible-light integrated optical phased arrays and visible-light-activated photochemistry; the system consists of a visible-light integrated optical phased array that emits and non-mechanically steers a beam up into a well of visible-light-curable resin. Third, we utilized this system to photocure a voxel, thus demonstrating 3D printing using a chip-based system for the first time. Fourth, we characterized the curing rate of this system by measuring the size of individual 3D-printed voxels as a function of curing time, observing printing of sub-millimeter-scale voxels within seconds. Fifth, we utilized the non-mechanical beam-steering capabilities of the system to implement 3D printing of lines in one dimension without any moving parts. Finally, we extended this capability to demonstrate 3D printing of arbitrary patterns in two dimensions using the system.

In the future, we will extend the preliminary proof-of-concept demonstration presented in this work and demonstrate the complete volumetric chip-based 3D printer concept. As an initial step utilizing our current chip-based 3D printer, we will couple an off-chip tunable visible-wavelength laser onto the chip, allowing us to perform non-mechanical wavelength-based beam steering also in the antenna dimension of the integrated optical phased array^66^. Additionally, we will modify a standard commercial SLA 3D printer by replacing the printer’s optics with our current chip; we will then use the commercial printer’s build platform and resin-coating mechanism to perform layer-by-layer 3D printing with the reduced optical and mechanical complexity offered by the non-mechanical beam-steering capabilities of our chip. Then, we will develop the next generation of the chip-based 3D printer based on a focusing integrated optical phased array^35^ to enable a novel demonstration of a completely non-mechanical 3D printer, in which high-resolution prints will be formed by a steerable focused beam with an adaptable focal height projected into a chamber of resin. Finally, we will demonstrate the proposed holographic optical-phased-array-based pixel architecture to implement a true single-shot volumetric 3D printing system, heterogeneously integrated with a co-designed CMOS electronics chip^30,33,34^, thus enabling solidification of entire 3D objects at once using a hand-held printer. In tandem with future iterations of the chip, we will also modify the visible-light-curable resins for compatibility with holographic 3D printing; specifically, we will work towards resins with decreased absorption and increased viscosity that operate via two-photon absorption^67^. Together, these additional innovations to both the chip and the resins will allow us to demonstrate the complete volumetric chip-based 3D printer vision.

The chip-based 3D-printing technology introduced in this work has the potential to enable a highly-compact, portable, and low-cost solution for the next generation of 3D printers. Such a solution would offer a more accessible and rapid mechanism for generating 3D objects, impacting a wide range of application areas, including military, medical, engineering, and consumer.

## Materials and methods

### Visible-light integrated optical phased array system fabrication

The liquid-crystal-based cascaded integrated optical phased array used in this work was fabricated in a CMOS-compatible 300-mm wafer-scale silicon-photonics process at the State University of New York Polytechnic Institute’s (SUNY Poly) Albany NanoTech Complex. The final fabricated cross section of the system, as received from SUNY Poly, consists of two 160-nm-thick SiN waveguiding layers separated by 250 nm of silicon dioxide, an 800-nm-deep and 5-µm-wide silicon-dioxide trench vertically spaced 60 nm above the top waveguiding layer, 820-nm-thick and 1-µm-wide metal electrodes on each side of the trench, and a smooth chip facet for edge coupling.

We then diced the fabricated photonics wafer and performed a chip-scale liquid-crystal-packaging process at MIT. This packaging process consists of four steps: (i) performing a dry etch to bring the trench closer to the waveguide; (ii) patterning an SU-8 photoresist spacer layer; (iii) epoxying a glass chip with an alignment layer on top of the SU-8 spacer layer; and (iv) injecting liquid crystal into the formed cavity, to achieve a final cross section as shown in Fig. 2c. Further details on the wafer fabrication and liquid-crystal packaging are provided in^55,68^.

### Visible-light-curable liquid resin synthesis

To create the visible-light-curable liquid resin used in this work, we first synthesized the photoredox compound (aza-Br) – an aza-boron-dipyrromethene (Aza-BODIPY) dye – using acetophenone and benzaldehyde derivatives as well as *N*-bromosuccinimide, in a process detailed in^46^. To complete the photosystem, we combine the aza-Br (7.6 mg, 0.4 mol%) with the coinitiator pair of Borate V (3.8 mg, 0.4 mol%) and H-Nu 254 (52.0 mg, 4.0 mol%). Finally, we dissolve the photosystem in a 10:1 mixture of tetraethylene glycol diacrylate (0.7 mL) and trimethylolpropane triacrylate (0.07 mL) to create the complete resin. We perform this process in a dark environment to avoid unnecessary onset of polymerization.

## Data Availability

Data is available upon reasonable request.
